# The Effects of Common Genetic Variation in 96 Genes Involved in Thyroid Hormone Regulation on TSH and FT4 Concentrations

**DOI:** 10.1210/clinem/dgac136

**Published:** 2022-03-09

**Authors:** Rosalie B T M Sterenborg, Tessel E Galesloot, Alexander Teumer, Romana T Netea-Maier, Doug Speed, Marcel E Meima, W Edward Visser, Johannes W A Smit, Robin P Peeters, Marco Medici

**Affiliations:** Department of Internal Medicine, Division of Endocrinology, Radboud University Medical Center, Nijmegen, The Netherlands; Academic Center for Thyroid Diseases, Department of Internal Medicine, Erasmus Medical Center, Rotterdam, The Netherlands; Department of Epidemiology, Erasmus Medical Center, Rotterdam, The Netherlands; Radboud University Medical Center, Radboud Institute for Health Sciences, Department for Health Evidence, Nijmegen, The Netherlands; Institute for Community Medicine, University Medicine Greifswald, Greifswald, Germany; DZHK (German Center for Cardiovascular Research), partner site Greifswald, Greifswald, Germany; Department of Population Medicine and Lifestyle Diseases Prevention, Medical University of Bialystok, Bialystok, Poland; Department of Internal Medicine, Division of Endocrinology, Radboud University Medical Center, Nijmegen, The Netherlands; Department of Quantitative Genetics and Genomics, Aarhus University, Aarhus, Denmark; Academic Center for Thyroid Diseases, Department of Internal Medicine, Erasmus Medical Center, Rotterdam, The Netherlands; Academic Center for Thyroid Diseases, Department of Internal Medicine, Erasmus Medical Center, Rotterdam, The Netherlands; Department of Internal Medicine, Division of Endocrinology, Radboud University Medical Center, Nijmegen, The Netherlands; Academic Center for Thyroid Diseases, Department of Internal Medicine, Erasmus Medical Center, Rotterdam, The Netherlands; Department of Internal Medicine, Division of Endocrinology, Radboud University Medical Center, Nijmegen, The Netherlands; Academic Center for Thyroid Diseases, Department of Internal Medicine, Erasmus Medical Center, Rotterdam, The Netherlands; Department of Epidemiology, Erasmus Medical Center, Rotterdam, The Netherlands

**Keywords:** thyroid, TSH, FT4, genetics, single nucleotide polymorphism, candidate gene

## Abstract

**Objective:**

While most of the variation in thyroid function is determined by genetic factors, single nucleotide polymorphisms (SNPs) identified via genome-wide association analyses have only explained ~5% to 9% of this variance so far. Most SNPs were in or nearby genes with no known role in thyroid hormone (TH) regulation. Therefore, we performed a large-scale candidate gene study investigating the effect of common genetic variation in established TH regulating genes on serum thyrotropin [thyroid-stimulating hormone (TSH)] and thyroxine (FT4) concentrations.

**Methods:**

SNPs in or within 10 kb of 96 TH regulating genes were included (30 031 TSH SNPs, and 29 962 FT4 SNPs). Associations were studied in 54 288 individuals from the ThyroidOmics Consortium. Linkage disequilibrium-based clumping was used to identify independently associated SNPs. SNP-based explained variances were calculated using SumHer software.

**Results:**

We identified 23 novel TSH-associated SNPs in predominantly hypothalamic-pituitary-thyroid axis genes and 25 novel FT4-associated SNPs in mainly peripheral metabolism and transport genes. Genome-wide SNP variation explained ~21% (SD 1.7) of the total variation in both TSH and FT4 concentrations, whereas SNPs in the 96 TH regulating genes explained 1.9% to 2.6% (SD 0.4).

**Conclusion:**

Here we report the largest candidate gene analysis on thyroid function, resulting in a substantial increase in the number of genetic variants determining TSH and FT4 concentrations. Interestingly, these candidate gene SNPs explain only a minor part of the variation in TSH and FT4 concentrations, which substantiates the need for large genetic studies including common and rare variants to unravel novel, yet unknown, pathways in TH regulation.

Thyroid hormones (TH) are essential for normal growth and differentiation, regulation of energy metabolism, and physiological function of virtually all human tissues (1, 2).

The hypothalamic-pituitary-thyroid (HPT) axis plays a predominant role in maintaining normal circulating TH concentrations. Hypothalamus derived thyroid-releasing hormone (TRH) stimulates the pituitary to release thyrotropin [thyroid-stimulating hormone (TSH)], which stimulates the thyroid to produce thyroxine (T4) and, to a lesser extent, triiodothyronine (T3). TH availability and action on a peripheral level is regulated by TH transporters, deiodinases, nuclear receptors, and receptor cofactors. TH degradation is primarily regulated by successive deiodination. Glucuronidation and sulfation result in elimination of TH via urine or feces or recycling in the enterohepatic circulation. All these biological processes influence the variation in TH concentrations.

In the last decade, epidemiological studies have demonstrated that subclinical thyroid disease, defined as TSH concentrations outside the reference range and free T4 (FT4) concentrations within the reference range, is associated with several adverse health outcomes, such as coronary heart disease, stroke, and atrial fibrillation, among others (3-5). Also, multiple studies have demonstrated that even subtle variation in thyroid function within the reference range associates with an increased risk of cardiovascular diseases, fractures, dementia, depression, and even mortality (4, 6-12). Some of these associations were found to be causal based on Mendelian randomization studies (13-16). Therefore, there is a high need to identify the factors responsible for these variations in thyroid function.

Twin studies have shown that the majority (up to 57-71%) of the interindividual variation in thyroid function is determined by genetic factors (17). In the last 20 years, several candidate gene studies have been performed, assessing the effects of genetic variation in known TH regulation genes on TSH and FT4 concentrations. However, most studies were limited by the assessment of only a few candidate genes and small sample sizes with low power to detect true-positive findings. Based on these studies, genetic variation in only a few candidate genes has been consistently associated with thyroid function, including the TSH receptor (*TSHR*), deiodinases type 1 and 2 (*DIO1*, *DIO2*) and TH transporters (*MCT8*, *OATP1B1*, and *OATP1C1*) (18-27).

Therefore, hypothesis-driven candidate gene approaches shifted toward large genome-wide association studies (GWAS). The latest and largest GWAS up to now identified 92 common (minor allele frequency > 1%) genetic variants associated with variation in normal range TSH and FT4 concentrations, which together only explained 21% to 33% of the common genetic variance in normal range thyroid function (28). Interestingly, many of the identified genes do not have a known role in TH regulation. Thus, the extent to which common genetic variation in known TH regulating genes contributes to thyroid function is still largely unknown.

For these reasons, we studied the role of single nucleotide polymorphisms (SNPs) in 96 established TH regulating genes on serum TSH and FT4 concentrations in up to 54 288 individuals from 22 European populations. To obtain a comprehensive overview of the current state of this field, we furthermore assessed which part of the variation in thyroid function is explained by variants identified with GWAS, common variants in all TH regulating candidate genes, and common variants in the entire genome.

## Material and Methods

### Candidate Gene Selection

**Figure 1.**
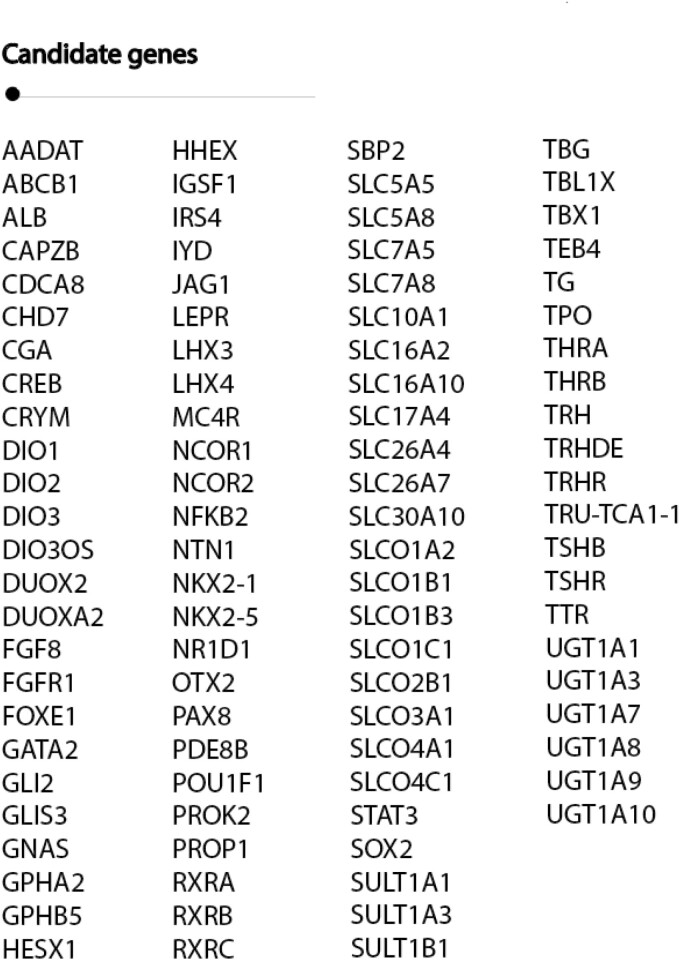
Candidate gene selection of genes involved in thyroid hormone regulation. Gene synonyms: DUOX2/THOX2, FOXE1/TTF2, IYD/DEHAL1, NCOR2/SMRT, NKX2-1/TTF1, RXRC/RXRG, SLC5A5/NIS, SLC5A8/SMCT, SLC7A5/LAT1, SLC7A8/LAT2, SLC10A1/NTCP, SLC16A2/MCT8, SLC16A10/MCT10, SLC26A4/PDS, SLCO1A2/OATP1A2, SLCO1B1/OATP1B1, SLCO1B1/OATP1B3, SLCO1C1/OATP1C1, SLCO2B1/OATP2B1, SLCO3A1/OATP3A1, SLCO4A1/OATP4A1, SLCO4C1/OATP4C1, SPB2/SECISBP2, TBG/SERPINA7, TEB4/MARCH6

A comprehensive search strategy in PubMed, was executed from inception to February 2021 limited by “humans,” “English,” and “review” to create a complete list of genes involved in TH regulation [Supplementary Figure 1, Supplementary Table 1 (29)]. Genes with a known role in TH synthesis, transport, metabolism, and signaling, as well as in congenital hypothyroidism and combined pituitary hormone deficiency, were included. All articles were screened for eligibility based on title and abstract. Abstracts of references were checked if titles were corresponding to the search strategy. After review by 3 independent experts in the field (M.E.M., M.M., W.E.V.), 96 genes with an established role in TH regulation were selected [Figure 1, Supplementary Table 2 (29)].

### ThyroidOmics Consortium Data Set

SNPs associations with thyroid function data were derived from the largest GWAS meta-analyses of normal range TSH and FT4 concentrations, performed by the ThyroidOmics Consortium (www.thyroidomics.com) (28). This study included 54 288 and 49 269 participants for TSH and FT4, respectively (discovery phase). All participants were of European ancestry, and only individuals with TSH concentrations within the cohort specific reference range were included for both TSH and FT4 analyses. Participants with non-European ancestry, thyroid medication use (Anatomical Therapeutic Chemical code H03), or those who underwent thyroid surgery were excluded.

As this candidate gene study focused on the effects of genetic variants on normal range thyroid function, we did not use the summary statistics of the published TSH GWAS by Zhou et al as their analyses were not restricted to individuals with normal range TSH concentrations (30).

### Statistical Analyses

We extracted association statistics of all SNPs present within the 96 candidate genes and 10 kb flanking region with a minor allele frequency > 1% from the ThyroidOmics Consortium data set. Testing thousands of SNPs in these 96 gene regions instead of millions of SNPs in the entire genome (TSH 30 031 SNPs, FT4 29 962 SNPs), a false discovery rate (FDR) of 1% proposed by Benjamini and Hochberg (p.adjust in R) was applied to correct for multiple testing (31, 32). This method reduces false positives, but also minimizes false negatives. To select independently associated SNPs for each trait, linkage disequilibrium (LD)-based clumping was performed in PLINK v2.0 on the results that passed the FDR using the 1000Genome phase 1v3 all ethnicities panel as a reference. Significance threshold filters (p1 and p2) were set to default 1 to include all SNPs in the clump (33). Specifically, this procedure keeps the most significant SNPs by region in the genome and removes SNPs that are too correlated (ie, no pair within 1 Mb had squared correlation > 0.01). SNPs that survived the clump were considered novel when these were previously not identified as index variants (genome-wide significant) or in LD (r2 > 0.01 within windows of ±1 Mb) with index variants in the normal thyroid function GWAS in which millions of SNPs are tested. To add on the robustness of the previous analysis, a secondary analysis using the conservative Bonferroni multiple testing correction (0.05/independent SNPs) was performed. To estimate the number of statistically independent SNPs in our candidate SNP selection, the indep option in PLINK was used. By this method, correlated SNPs (based on a multiple correlation coefficient and the LD) were removed. To remove highly correlated SNPs from our selected gene regions, a 2000 kb window shifted by 5 consecutive SNPs and a variation inflation factor of 5 corresponding to a multiple correlation coefficient R² = 0.8 for a SNP being regressed on all other SNPs simultaneously were selected as parameters (33). If the GWAS *P*-value of the SNP was <0.05/independent SNPs, the SNP passed Bonferroni correction. To assess the effect sizes of the associated independent SNPs on both TSH and FT4 concentrations, beta-beta plots were provided [Supplementary Figures 2 and 3 (29)].

### SNP-based Explained Variance

We calculated SNP-based heritabilities for normal range TSH and FT4 based on the ThyroidOmics Consortium summary statistics using the LDAK-thin model and BLD-LDAK model as implemented in SumHer software (28, 34, 35). As a reference panel, we used genome-wide SNP data from the Nijmegen Biomedical Study (NBS; n = 4664), which has the same ancestry as the cohorts included (ie, European), as NBS was also included in this meta-GWAS (36). In addition to the overall SNP-based heritability of TSH and FT4 concentrations, explained variances for the following sets of SNPs were calculated: (1) known TSH GWAS SNPs, (2) novel independent TSH SNPs from our current candidate gene analysis, (3) known and novel TSH SNPs in the selected candidate genes (sets 2 and 4 combined), (4) known TSH GWAS SNPs in the selected candidate genes, (5) known TSH GWAS SNPs outside of the selected candidate genes, (6) known FT4 GWAS SNPs, (7) novel FT4 SNPs from our current candidate-gene analysis, (8) known and novel FT4 SNPs in the selected candidate genes (sets 7 and 9 combined), (9) known FT4 GWAS SNPs in the selected candidate genes, (10) known FT4 GWAS SNPs outside of the selected candidate genes, and (11) all SNPs in all 96 candidate genes + 10 kb flanking region. For SNPs that were not present in our reference, we selected a LD proxy if available with an r^2^ value (correlation) of at least 0.8 according to 1000 Genomes data from the CEU population as reported in the LDlink database (https://ldlink.nci.nih.gov/) [Supplementary Table 3 (29)]. Extended method description can be found in Supplementary Methods (29). Results of the model with the lowest Akaike information criterion were used.

## Results

Figure 1 shows all selected TH candidate genes. Baseline characteristics of the ThyroidOmics Consortium data set are displayed in Table 1.

**Table 1.** Baseline characteristics of the normal thyroid function

Characteristic	
Participants (n)	
TSH	54 288
FT4	49 269
Total cohorts (n)	
TSH	22
FT4	19
Ethnicity	Caucasian
Age, years	55.1 (11.8)
Female, %	52.0
TSH, mU/L	1.80 (1.88)
FT4, pmol/L	14.9 (2.4)

GWAS characteristic data are given as n or pooled mean (SD).

Abbreviations: FT4, free thyroxine; TSH, thyroid-stimulating hormone (thyrotropin).

### Genetic Variation in Candidate Genes and Normal Range TSH Concentrations

**Figure 2.**
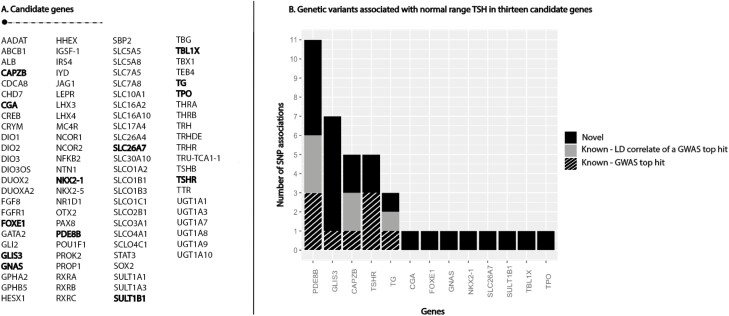
Independent genetic variants associated with normal range thyrotropin [thyroid-stimulating hormone (TSH)] in 13 candidate genes. (A) List of included thyroid hormone-regulating candidate genes. Genes with at least 1 independent signal are depicted in bold. (B) The y-axis shows the number of independently associated genetic variants with serum normal range TSH concentrations per gene (x-axis). Only genes with at least 1 significant single nucleotide polymorphism (SNP) association are included. The novel variants are depicted in black, linkage disequilibrium (LD) correlated variants with a genome-wide association study (GWAS) loci outside the candidate gene region in grey and known top hits from GWAS are depicted in a striped pattern.

Thirty-nine SNPs in or nearby 13 candidate genes were independently associated with TSH concentrations. Of these, 23 SNPs in 13 genes were novel [Fig. 2; also see Supplementary Table 4 (29)] (28). Novel genes included genes important for TSH signalling (*CGA*, *GNAS*, *TBL1X*), regulation of thyroid differentiation and expression or transcription of thyroid-specific genes (*FOXE1*, *NKX2-1*), transportation of iodide into the thyroid and iodination of tyrosine residues (*SLC26A7*, *TPO*) and TH conjugation (*SULT1B1*). The functions of all candidate genes that contained at least one statistically significant association signal are described in Supplementary Table 5 (29). The candidate genes that showed the highest number of statistically significant novel independent signals were *GLIS3* (6 SNPs) and *PDE8B* (5 SNPs), which both play a central role in the HPT axis. Ten of the 23 novel SNPs passed a stricter Bonferroni multiple testing correction (0.05/1615 independent SNPs) in the *CAPZB*, *CGA*, *FOXE1*, *GLIS3* (n = 2), *PDE8B* (n = 3), *TPO*, and the *TSHR* genes [Supplementary Table 4 (29)].

### Genetic Variation in Candidate Genes and FT4 Concentrations

**Figure 3.**
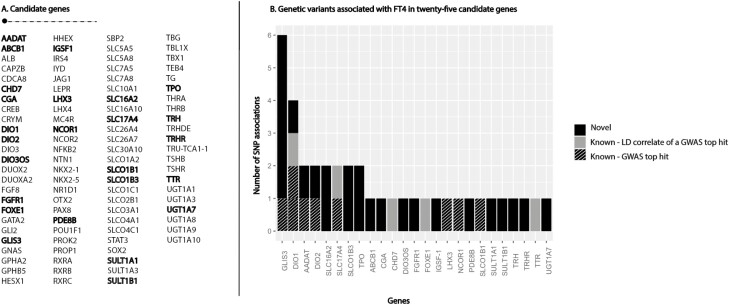
Independent genetic variants associated with free thyroxine (FT4) in 25 candidate genes. (A) List of included thyroid hormone-regulating candidate genes. Genes with at least 1 independent signal are depicted in bold. (B) The y-axis shows the number of independently associated genetic variants with serum FT4 concentrations per gene (x-axis). Only genes with at least 1 significant single nucleotide polymorphism (SNP) association are included. The novel variants are depicted in black, linkage disequilibrium (LD) correlated variants with a genome-wide association study (GWAS) loci outside the candidate gene region in grey and known top hits from GWAS are depicted in a striped pattern.

Thirty-nine independent genetic variants in or nearby 25 candidate genes were associated with FT4. Of these, 25 variants in 18 candidate genes were novel [Fig. 3; also see Supplementary Table 6 (29)] (28). The functions of the 25 candidate genes are described in Supplementary Table 7 (29). Novel associated candidate genes are genes important in TH metabolism (*DIO3OS*, *SULT1A1*, *SULT1B1*, *UGT1A7*), TH transport (*ABCB1*, *SLC16A2*, *SLCO1B1*), regulation of peripheral TH action (*NCOR1*), encoding or influencing the expression of central players in the HPT axis (*TRH*, *TRHR*, *IGSF-1*, *CGA*, *PDE8B*) or important in pituitary development and differentiation (*LHX3*, *FGFR1*). The candidate genes that showed the highest number of novel statistically significant independent signals were *GLIS3* (5 SNPs), *SLC16A2* (encoding the MCT8 transporter; 2 SNPs), *SLCO1B3* (2 SNPs), and *TPO* (2 SNPs). Fifteen of the 25 novel SNPs passed Bonferroni correction (0.05/1623 independent SNPs) in, respectively, the *AADAT*, *CGA*, *DIO2*, *DIO3OS*, *GLIS3* (n = 3), *IGFS-1*, *PDE8B*, *SLC16A2*, *SLCO1B3*, *SULT1A1*, *TPO*, *TRH*, and *TRHR* genes [Supplementary Table 6 (29)].

### Limited Overlap Between TSH and FT4

Six candidate genes—namely, *GLIS3*, *CGA, FOXE1*, *PDE8B*, *SULT1B1*, and *TPO*—included SNPs that were either associated with TSH or FT4 concentrations, while none of these SNPs were associated with both TSH and FT4 concentrations. Among these, SNPs in the *GLIS3* and *PDE8B* genes have previously been associated with TSH (28). The FT4-associated SNP in *FOXE1* is in LD with a genome-wide significant variant allocated to the nearest gene *FOXE1* (*rs10739496*), which falls outside the candidate gene region used in this study (28). All TSH-associated independent SNPs showed larger effect sizes on TSH compared to the effect on FT4 concentrations [Supplementary Figure 2 (29)]. For FT4, only 1 SNP showed a larger effect on TSH compared to FT4 (*PDE8B* gene), while the others showed all larger effects on FT4 compared to TSH concentrations.

### SNP-Based Heritability

**Figure 4.**
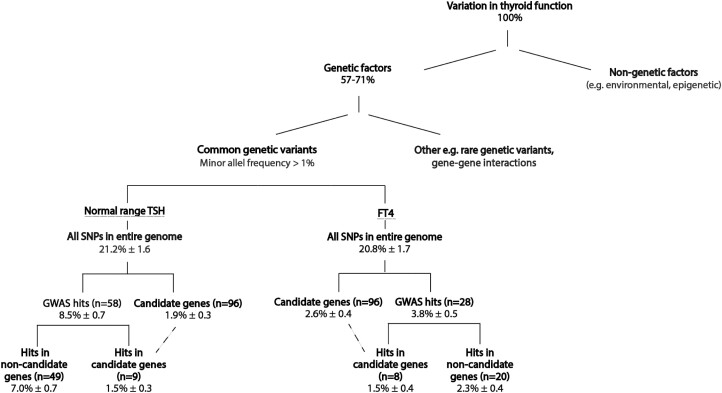
Current state of knowledge of the variation in normal range thyroid function explained by genetic factors. The estimate of variance explained by genetics (57-71%) is based on twin studies (17). All other depicted explained variances are calculated using the BLD-LDAK model in SumHer software and are displayed as heritability mean ± SD (34, 35). The dashed line indicates the overlap in explained variance of the hits identified in genome-wide association study candidate genes. Abbreviations: FT4, free thyroxine; GWAS, genome-wide association study; SNP, single nucleotide polymorphism; TSH, thyroid-stimulating hormone.

All common SNPs (minor allele frequency > 1%) across the entire genome explained 21.2% (SD 1.64) and 20.8% (SD 1.73) of the variance in both TSH and FT4 serum concentrations, respectively. All common SNPs in or near all 96 candidate genes explained 1.9% (SD 0.35) of the variation in TSH and 2.6% (SD 0.41) in FT4 concentrations. Among these, the statistically significantly associated TSH and FT4 SNPs only explained 1.8% (SD 0.35) and 2.1% (SD 0.41) of the variation in TSH and FT4 concentrations, respectively. Figure 4 provides a comprehensive overview of the heritability of normal thyroid function, including the addition of currently known GWAS variants. Supplemental Table 8 provides an overview of calculated explained variances for all gene subsets based on the BLD-LDAK model (29).

## Discussion

Here, we present the results of the largest candidate gene study on genetic determinants of thyroid function. With 23 newly associated variants for TSH and 25 variants for FT4, this approach resulted in a substantial increase in the number of variants known to determine interindividual variation in thyroid function. Among the TSH associated variants, an overrepresentation was seen for genes with a central role in the HPT axis (*TSHR*, *TPO*, *PDE8B*, *CAPZB*, *FOXE1*) [Supplementary Table 5 (29)]. In contrast, among the FT4-associated variants, there is an overrepresentation of genes implicated in local TH bioavailability, including genes involved in TH metabolism [ie, the deiodinases (*DIO1*, *DIO2*, *DIO3OS*) and *AADAT*], several transporters (*SLC16A2*, *SLC17A4*, *SLCO1B1*, *SLCO1B3*), and genes involved in conjugation by sulfation (*SULT1A1*, *SULT1B1*) and glucuronidation (*UGT1A7*) [Supplementary Table 7 (29)]. The role of these genes in determining local (and not central) TH bioavailability is also supported by the observation that these FT4-associated variants were not associated with TSH concentrations. This is supported by the beta-beta plots showing that the vast majority of independently associated genetic variants have larger effect sizes on either TSH or FT4 [Supplementary Figures 2 and 3 (29)].

Several genes showed SNP associations with both TSH and FT4. Associations with *GLIS3* (TSH, FT4), *PDE8B* (TSH), and FOXE1 (FT4) have been identified in a previous GWAS (28), playing an important role in either thyroid development, growth and differentiation (*GLIS3*, *FOXE1*), TSH signaling (*PDE8B*), or TSH-regulated gene expression (*FOXE1*). The relation with both TSH and FT4 for *GLIS3* has been described in literature, which is based on rare mutations leading to congenital hypothyroidism (37, 38). Common genetic variants in *PDE8B,* affecting 3′,5′-cyclic adenosine 5′-monophosphate concentrations in the thyroid, have also shown associations with reciprocal changes in TSH and suggestive associations with FT4 concentrations (39).

The *FOXE1* gene is under tight control of TSH, regulating transcription of several genes important in thyrocyte function. Mutations in this gene can lead to primary congenital hypothyroidism affecting both TSH and FT4 concentrations (40).

More interestingly, *SULT1B1*, encoding a sulfotransferase enzyme catalyzing the sulfate conjugation of many hormones, including TH, also showed associations with TSH or FT4. Primarily, THs are degraded through successive iodination. Subsequent conjugation of TH in the liver or kidney by either glucuronidation or sulfation will increase the water solubility, resulting in either secretion in the bile or further deiodination if sulphated. Whereas glucuronidated TH may be recycled in the enterohepatic circulation, conjugation will lead to its final elimination in urine or feces. Therefore, the relation with FT4 can be explained by a direct effect on TH degradation, while the effect on TSH can be explained via the negative feedback loop of the HPT axis, aimed at maintaining normal circulating TH concentrations.

SNPs associated with TSH in *CGA* (rs6924373) and *TPO* (rs9678281) showed genome-wide significance after replication in a candidate gene approach based on orthologous genes causing abnormal thyroid physiology in mice (28). *TPO* encodes thyroid peroxidase, which is important for the biosynthesis of TH catalyzing the iodination of tyrosines on thyroglobulin. *CGA* encodes, among others, the alpha chain of TSH. In our study, these 2 associations with TSH were considered as novel, as these SNPs did not reach genome-wide significance in the discovery phase of the mentioned GWAS.

Next to identifying novel SNP associations, we provided an overview of the explained variances of TSH and FT4 by genetic factors, as calculated by the BLD-LDAK model using summary statistics. In previous published studies, different methods for calculating the explained variance have been used, such as GCTA and LDSC using individual-level data or a simplified equation, which takes the beta and allele frequency into account. SNP heritabilities cannot be compared between studies when different calculation methods have been used. Therefore, we used the same model with the similar reference (ie, NBS) in this study when calculating explained variances for the different SNP sets.

Despite the identification of a high number of novel genetic variants, our analyses showed that the common variants in the TH regulating genes examined in this study explain 1.9% (TSH) to 2.6% (FT4) of the variance in normal thyroid function (Fig. 4), while all common SNPs in the genome explain ~21%. This strongly suggests that other, yet unknown, pathways are involved in TH regulation. Indeed, most of the genetic variants identified in TSH and FT4 GWAS are located in loci with a yet unknown role in TH regulation, namely 7.0% (SD 0.68) for TSH and 2.3% (SD 0.4) for FT4 (Fig. 4). The potential gains of further functional in vitro studies are illustrated by a previous GWAS on thyroid function, in which *AADAT* was identified as a novel metabolizing enzyme and *SLC17A4* as a novel TH transporter (28). Next to novel pathways directly involved in TH regulation, it could be speculated that part of the yet unknown pathways involve genes important for the development and homeostasis of specific cell structures or cell populations in the HPT axis. Gene-enrichment testing as well as pathway analyses with novel GWAS data on thyroid function could answer these questions.

Next to variants in novel pathways, part of the missing heritability of thyroid function is likely explained by rare variants with larger effect sizes, as current GWAS markers typically do not tag most rare variants well. In recent years, it has also been shown for other complex traits, such as irritable bowel syndrome, type 2 diabetes, and coronary artery disease, that rare variants play an important role in the explained genetic variance (41-44). For thyroid function, only 1 whole-genome sequencing study (n = 2287) has been performed yielding mainly novel common variants (45). It is therefore key that future studies focusing on rare genetic variants are sufficiently powered.

Strengths of the current study include the large sample size, the thorough selection of a comprehensive list of established TH regulating genes, and the use of a multiple testing correction in combination with a stringent LD threshold for clumping. Furthermore, we added a sensitivity analysis using the conservative Bonferroni multiple testing correction, which takes the number of independent SNPs into account. This minimalized the chance of false-positive findings, hereby taking the limitations of previous candidate gene studies into account. The results demonstrate that the Bonferroni correction extracts fewer but likely more robust true-positive findings. However, it is commonly accepted to use the FDR in genetic studies, which also corrects for the type 1 error (32). Furthermore, it can be speculated whether the Bonferroni correction would be too conservative for this study, given the preselection of genes with a known role in or associated with TH regulation. For completeness, we showed both FDR and Bonferroni corrected outcomes. Future larger GWAS could provide clarity regarding reproducibility of the current findings using FDR.

This study has also some limitations. Currently, the vast majority of studies have assessed genetic associations with thyroid function in populations of European descent (46). Our findings should therefore not be extrapolated to non-European populations. Although our findings were derived from large meta-analyses of up to 22 cohorts and by applying correction for multiple testing, there is still a chance that at least some of the results are false positive. Given the large sample sizes used in this study, we were not able to confirm our findings by performing a replication analysis in a well-powered, independent cohort. However, with the sensitivity analyses we showed that 67% to 74% of the SNPs identified with FDR survives conservative multiple testing for familywise error rate, providing an extra layer of evidence that these SNPs in candidate genes are important determinants of the variation in thyroid function. Furthermore, as no large-scale data sets are yet available for serum T3 concentrations, we were unable to investigate effects on serum T3 concentrations. Finally, not all SNPs in the different subsets were present in the NBS reference sample; hence, the SNP-based explained variances for the different subsets might be slightly underestimated [Supplementary Table 3 (29)].

In summary, we provided a comprehensive overview of the effects of common genetic variation in TH regulating genes on thyroid function. While this approach yielded a number of novel independently associated variants, we also showed that genetic variation in the currently known TH regulating genes only explains a remarkable small part of the variation in thyroid function. Therefore, large-scale genetic studies focusing on both common and rare variation are needed, together with in vitro studies, to identify yet unknown pathways and elucidating the missing heritability in thyroid function.

## Data Availability

Some or all data generated or analyzed during this study are included in this published article or in the data repositories listed in the references (29). Please download the Supplementary Table file in the data repository for optimal view.
